# The Effects of Fermentable Oligo‐, Di‐, and Monosaccharides and Polyols on Dyspeptic Symptoms: A Systematic Review and Meta‐Analysis of Clinical Trials

**DOI:** 10.1002/hsr2.72662

**Published:** 2026-06-18

**Authors:** Seyedeh‐Zeynab Hosseinian, Fahimeh Haghighatdoost, Parisa Hajihashemi, Peyman Adibi

**Affiliations:** ^1^ Isfahan Gastroenterology and Hepatology Research Center Isfahan University of Medical Sciences Isfahan Iran; ^2^ Isfahan Cardiovascular Research Center, Cardiovascular Research Institute Isfahan University of Medical Sciences Isfahan Iran; ^3^ Nutrition and Food Security Research Center Isfahan University of Medical Sciences Isfahan Iran

**Keywords:** bloating, dyspeptic symptoms, early satiety, FODMAPs, meta‐analysis

## Abstract

**Background and Aims:**

Conflicting evidence is available regarding the effects of fermentable oligo‐, di‐, and monosaccharides and polyols (FODMAP) on dyspeptic symptoms. Therefore, this systematic review and meta‐analysis was performed to assess the effects of FODMAPs on dyspeptic symptoms.

**Methods:**

A systematic search up to October 2025 was performed through PubMed, ISI Web of Science, Scopus, Cochrane Library, and EMBASE. Randomized controlled trials (RCTs) and non‐randomized studies of interventions (NRSIs) examined the impact of the low‐FODMAP diet (LFD) as the intervention group and the high‐FODMAP diet (HFD) as the control diet on dyspeptic symptoms, including early satiety, pain, bloating, fullness, and nausea were included. The search strategy involved combining FODMAP‐related terms with dyspeptic terms and symptoms. The mean severity of dyspeptic symptoms was pooled using a random‐effects model and was reported as the weighted mean difference (WMD) and 95% confidence interval (CI). The certainty of the evidence was assessed with the Grading of Recommendations Assessment, Development and Evaluation (GRADE).

**Results:**

A total of 12 studies (6 RCTs and 6 NRSIs) were eligible for inclusion in this meta‐analysis. Compared with an HFD, an LFD significantly decreased the severity of bloating (WMD = −0.36, 95% CI: −0.58, −0.14; *I* ^2^ = 88.3%, *n* = 8) and early satiety (WMD = −0.69, 95% CI: −0.92, −0.46; *I* ^2^ = 86.3%, *n* = 8). However, compared with an HFD, an LFD was associated with greater abdominal pain scores (WMD = 0.34, 95% CI: 0.11, 0.56; *I* ^2^ = 34.5%, *n* = 7) but did not influence nausea (WMD = 0.12, 95% CI: −0.09, 0.32; *I* ^2^ = 0.0%, *n* = 7) or fullness severity (WMD = −1.63, 95% CI: −2.92, −0.35; *I* ^2^ = 98.4%, *n* = 3).

**Conclusion:**

An LFD significantly reduced the severity of bloating and early satiety. However, these findings were substantially heterogeneous, and the certainty of the evidence was very low, which needs to be confirmed by further RCTs.

## 1 Introduction

Dyspeptic symptoms primarily originate from the gastroduodenal region and include epigastric pain, epigastric burning, postprandial fullness, early satiety, nausea, and bloating [1]. Despite routine clinical evaluation, these symptoms often remain unexplained, leading to the diagnosis of functional dyspepsia (FD), a widely recognized and prevalent functional gastrointestinal disorders (FGIDs) [1, 2]. Dyspeptic symptoms have a substantial impact on quality of life and impose a significant economic burden [3]. Although the underlying causes of dyspeptic symptoms remain uncertain, several factors, such as dysfunction in the gut‐brain axis, visceral hypersensitivity, altered gut motility, disrupted mucosal function, and imbalanced gut microbiota, have been implicated [2].

Dietary factors have been recognized as contributors to dyspeptic symptoms, with many patients reporting a relationship between certain foods and their symptoms [2]. Notably, high‐fat foods, wheat‐based products, carbonated drinks, fruits, milk, processed foods, and pepper have been suggested as potential triggers [4, 5, 6]. Conversely, dietary restriction approaches such as low‐FODMAP (fermentable oligo‐, di‐, monosaccharides, and polyols) diet (LFD), and gluten‐free diet (GFD) have shown promise in alleviating dyspeptic symptoms [7]. FODMAPs, which are poorly absorbed in the gastrointestinal (GI) lumen, can contribute to increasing osmotic activity, fermentation, and gas production and consequently triggering GI symptoms [8]. Therefore, an LFD has been proposed for managing GI symptoms [9]. An LFD consists of three phases in which FODMAPs are eliminated for 6 to 8 weeks in the first step, and then, if symptoms are improved, specific FODMAPs are reintroduced to identify triggers. The final step is establishing a long‐term, personalized FODMAP diet where only trigger foods are eliminated [9]. The first phase of LFD has been shown to reduce symptoms in patients with irritable bowel syndrome (IBS) [10, 11]. Although FD and IBS are both common FGIDs, they affect different parts of the GI tract and present with different symptom patterns, which require distinct clinical approaches and therapeutic strategies [4]. However, considering that FGIDs share overlapping pathophysiological mechanisms [12], and that symptoms of FD frequently coexist with IBS symptoms [13], an LFD may have potential benefits in alleviating dyspeptic symptoms as well.

Previous research, including a systematic review of four interventional and two cross‐sectional studies on patients with FD, indicates insufficient evidence to support the effectiveness of an LFD for these patients [14]. Due to the limited literature, it is helpful to also consider studies involving other nonstructural conditions or healthy individuals to better understand LFD's impact on dyspeptic symptoms. Despite few publications, conducting a new systematic review is essential to include these additional studies. Furthermore, there is no clear consensus on LFD's effects; some studies report reduced early satiety after LFD [15, 16], while others do not [17, 18], with similar conflicting results for symptoms like fullness and bloating [15, 16, 18, 19]. Given the lack of comprehensive pooled data, a meta‐analysis is needed to thoroughly assess the current evidence on FODMAPs and dyspeptic symptoms.

In this study, we aimed to provide a systematic review and meta‐analysis of RCTs and NRSI assessing the effect of FODMAPs on dyspeptic symptoms including early satiety, pain, bloating, fullness, and nausea in adult subjects irrespective of the participant's health status.

## 2 Methods

The current systematic review and meta‐analysis were conducted based on the preferred reporting items for systematic review and meta‐analysis (PRISMA) guidelines [20].

### 2.1 Search Strategy

Relevant studies published up to 6 June 2023 were searched systematically through the electronic databases of PubMed, ISI Web of Science, Scopus, Cochrane Library, and EMBASE with no restrictions on publication date or language. The search strategy involved combining FODMAP‐related terms (fodmap, fermentable poorly absorbed short‐chain carbohydrate, fermentable carbohydrate) with dyspeptic terms and symptoms (Dyspep*, indigestion, indigestive, NUD, epigastric pain, epigastric burn, early satiety, early satiation, postprandial pain, postprandial distress, postprandial burn, postprandial fullness, gastrointestinal symptoms, functional bowel disorder, functional gastrointestinal disorder, pain, bloating, nausea). The reference lists of the retrieved articles were also examined to avoid missing any publications. The search was updated until October 2025.

### 2.2 Study Selection

The inclusion criteria were as follows: (1) Original clinical trials including randomized controlled trials (RCTs) and non‐ randomized studies of interventions (NRSI), with at least a NRSI that includes before‐and‐after data. The inclusion of studies will be based on an independent evaluation of their design, rather than the study labeling [21]. (2) Adult participants (aged ≥ 18), including healthy individuals or patients with any GI symptom without any structural health condition. (3) Dietary intervention with reported FODMAP content, where the LFD was defined as dietary advice or consumption of specific low FODMAP items or meals, and the high‐FODMAP diet (HFD) as dietary advice without FODMAP limitations or consumption of food items or meals with higher FODMAPs components. In RCTs, we did not assign significant importance to specific labels used in the original articles, such as intervention or control. Instead, we considered the LFD as the intervention and HFD as the control. (4) Utilizing validated patient‐reported outcome measures (PROMs) for FD, researcher‐designed questionnaires with related similar symptom profiles, or culturally adapted measures [22]. For instance, the pain domain of the Gastrointestinal Symptom Rating Score (GSRS) questionnaire, which includes abdominal pain, bloating, and nausea, was considered valid and reliable for assessing symptoms of FD [23]. Global FD syndrome was defined based on specific criteria or a clinician's opinion, as well as dyspeptic symptoms (including fullness, epigastric bloating, early satiety, epigastric pain, epigastric burning, epigastric cramp, epigastric discomfort, nausea, and dyspepsia) assessed by patient‐reported questionnaires.

Studies were excluded if they involved children, adolescents, participants with structural GI diseases (e.g., celiac disease and inflammatory bowel disease), or those who received other interventions alongside FODMAP. Additionally, commentaries, editorials, reviews, animal or in vitro studies, unpublished articles, and gray literature were excluded.

According to the Cochrane Handbook (section 24.1), NRSIs are defined as studies estimating the effect of an intervention without using randomization (e.g., during usual treatment decisions). These studies are often labeled as observational studies, provide results “in absolute terms” and are reviewed when RCTs cannot adequately answer the research question [24]. While they do not directly compare new treatments to existing ones, they offer several advantages: supporting marketing authorization, reimbursement, or practice changes; being quicker and more cost‐effective; identifying potential therapeutic effects and adverse reactions; and providing crucial initial evidence of efficacy and safety for early‐stage treatments and diets [21].

### 2.3 Data Extraction and Quality Assessment

Two reviewers (S.Z.H., P.H.) independently screened titles and abstracts to identify eligible studies and then assessed the full texts using the PICOS criteria. The authors extracted data on the first author's last name, publication year, country, trial design, participants' characteristics (number, age, sex), intervention and control diet, duration of intervention, outcome measures, and relevant results. If a study's primary intervention was an HFD with the control being an LFD, we reversed their roles, considering the LFD as the intervention and the HFD as the control. Two independent reviewers (S.Z.H., F.H.) assessed the quality of the RCTs using the Cochrane Collaboration Risk of Bias Tool [25]. Selection, performance, attrition, reporting, and detection bias were evaluated to judge studies as having low, high, or unclear risk of bias. NRSIs were assessed using the Risk of Bias In Nonrandomized Studies of Interventions (ROBINS‐I) tool [26], covering confounders, participant selection, intervention classification, deviations from intended interventions, missing data, outcome measurement, and selection of reported results [26]. Based on the Cochrane Handbook part 24.5.1, NRSIs have different abilities to show causal effect. Their biases depend on their specific design features, which can make some studies stronger and others weaker [24]. Discrepancies were resolved through discussion with the principal investigator (P.A.).

The 16‐item AMSTAR 2 tool was used to assess the methodological quality of the systematic review. Adherence was rated as “yes,” “partial yes,” or “no,” indicating high, moderate, low, or critically low quality, respectively [27]. The Grading of Recommendations, Assessment, Development, and Evaluation (GRADE) approach evaluated the quality of evidence and strength of recommendations, classifying them as high, moderate, low, or very low.

### 2.4 Meta‐Analysis

Analyses were conducted separately for RCTs and NRSIs to estimate mean differences in severity scores of dyspeptic symptoms. Only symptoms with sufficient data across studies were included. Statistical parameters, such as the mean and standard deviation (SD), were collected for baseline, endpoint, and changes from baseline. Data reported as the median and interquartile range (IQR), 95% confidence interval (CI), or standard error of the mean (SEM) were converted to mean and SD [28]. If necessary, data were not provided, attempts were made to contact the corresponding author. If no response was received, data were extracted from figures using WebPlotDigitizer [29] or the study was excluded. Results for different participant groups (e.g., responders and nonresponders) were combined using a formula from the Cochrane Handbook for Systematic Reviews of Interventions, part 6.5.2.10 [28].

Pooled estimates with 95% CIs were calculated using the random effects model to account for variation between studies [30]. Between‐studies heterogeneity was tested using *I* ^2^, with values greater than 50% indicating substantial heterogeneity [31]. Publication bias was evaluated using Egger's and Begg's tests [32, 33]. Sensitivity analysis was conducted using the leave‐one‐out method to examine the impact of each specific study on the pooled estimates. All analyses were performed using Stata 11.0 software (Stata Corp., College Station, TX, USA), with *p* values < 0.05 considered statistically significant.

## 3 Results

### 3.1 Search Results and Study Selection

**Figure 1:**
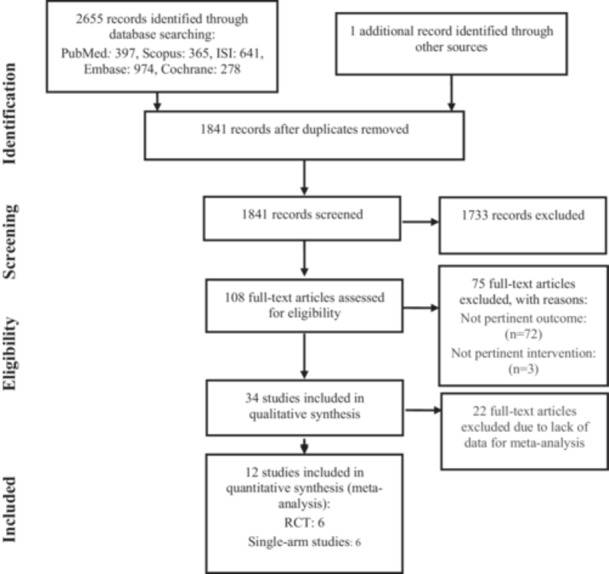
Flow chart of the studies reviewed.

The search identified 3168 studies at the initial stage (PubMed: 450, Embase: 1061, Scopus: 623, ISI: 716, Cochrane: 318). After duplicate removal and screening of 2451 articles, 113 full texts were assessed, 79 of which were excluded due to irrelevant outcomes (*n* = 77) or interventions (*n* = 3). Finally, 33 articles with 44 intervention arms were included in the systematic review (15 RCTs and 18 NRSIs). Meta‐analysis was performed using six RCTs and six NRSIs that had sufficient data for statistical analysis (Figure 1).

### 3.2 Study Characteristics

The characteristics of the 15 included RCTs (20 intervention arms) are summarized in Table 1. Nine studies had crossover designs, and six had parallel designs. The studies were conducted on patients with FD [15, 17], gastroesophageal reflux disease (GERD)‐like symptoms and IBS [16, 19], IBS [18, 35, 37, 40, 41, 42, 43], IBS and self‐reported nonceliac wheat sensitivity (NCWS) [39], self‐reported NCWS [38], GERD [34], and healthy individuals [36, 43]. Sample sizes ranged from 7 to 105 subjects. All RCTs were performed on both genders except for one, which was conducted on females [37]. Participants were aged 22 years and older. Intervention duration varied from 1 meal [36] to 8 weeks [15]. Approaches to prescribing LFDs included a feeding trial with provided low‐FODMAPs food [43], dietician‐led LFD advice in seven studies [15, 17, 34, 35, 40, 42], and specific foods like high‐FODMAP noodles [16, 18, 19], low‐FODMAP bread [37, 39, 41], fructans bars [38], and high‐calorie meal with 40 g of fructose or fructans [36].

**Table 1:** Characteristics of the RCTs included in the systematic review.

First author Year Location	Study design	Population (diagnostic criteria)	Number (intervention/control) female (%) age^a^ (y)	Intervention	Comparison	Duration (week, day, hour)	Dietitian involvement	Adherence monitoring	Outcome (outcome measurement tool)	Result	Source of funding	Conflict of interest
Goyal [15] 2022 India	Single‐blind Parallel RCT	Patients with FD (Rome IV criteria)	105 (54/51) 41% 18–70/37.6 ± 11.9	LFD advice	TDA advice	4 weeks	Yes	Daily food intake diary	Early satiety, fullness, epigastric pain, epigastric burning, epigastric discomfort, epigastric cramps, nausea, and bloating severity score based on SF‐NDI The proportion of responders with ≥ 50% reduction in SF‐NDI score The proportion of patients with adequate symptom relief	Early satiety, fullness, and, bloating severity scores were significantly lower after the intervention compared to the control (*p* = 0.0001, 0.002, 0.006). Epigastric pain, epigastric burning, epigastric discomfort, epigastric cramps, and nausea were lower but not significantly after the intervention compared to the control (*p* > 0.05). The proportion of responders was higher but not significantly in LFD compared to TDA (70.6% and 61.7%; *p* = 0.397)^d^. The proportion of patients with adequate symptom relief was higher but not significantly after LFD compared to TDA (62.9% and 50.9%; *p* = 0.24)^d^	None	None
Goyal [15] 2022 India	Single‐blind parallel RCT	Patients with FD improved on an LFD before the challenge. (Rome IV criteria)	105 (54/51) 41% 18–70/37.6 ± 11.9	LFD advice	TDA advice	8 weeks	Yes	Daily food intake diary	Early satiety, fullness, epigastric pain, epigastric burning, epigastric discomfort, epigastric cramps, nausea, and bloating severity score based on the symptom checklist of the SF‐NDI The proportion of responders with ≥ 50% reduction in SF‐NDI score	Early satiety, fullness, and bloating severity scores were significantly lower after the intervention compared to the control (*p* = 0.01, 0.001, 0.004). Epigastric pain, epigastric burning, epigastric discomfort, epigastric cramps, and nausea were lower but not significantly after the intervention compared to the control (*p* > 0.05). The proportion of responders was higher but not significantly after the intervention compared to the control (46.3% and 41.2%; *p* = 0.69).	None	None
Plaidum [16] 2022 Thailand	Single‐blind crossover RCT	Patient with GER and nonconstipation IBS (Rome III criteria)	8 (8/8) 75% 18–65/57 ± 13	Low FODMAPs rice noodle	High FODMAPs wheat noodle	1 day (breakfast and lunch) Washout: 1 week	NR	NR	Abdominal pain, bloating, nausea/vomiting, and early satiety severity scores (10 cm VAS).	Bloating and early satiety severity scores were significantly lower after the intervention compared to the control (*p* < 0.05). Abdominal pain and nausea/vomiting severity scores were not significantly lower after the intervention compared to the control.	The Ratchadapiseksompotch Fund, Chulalongkorn University, The Royal College of Physicians of Thailand (RCPT), and The Gastroenterological Association of Thailand (GAT).	None
Staudacher [17] 2021 Australia	Single‐blind parallel RCT	Patients with FD and coexisting IBS (SAGIS score)	59 (40/19) 78% > 18/46 ± 15	LFD advice	Standard dietary advice	NR	Yes	Judgment based on clinical notes	Postprandial pain, bloating, epigastric pain, abdominal cramps, early satiety, fullness, and nausea severity scores (5‐point scale). The proportion of responders with ≥ 30% reduction in SAGIS‐epigastric pain score	Postprandial pain and bloating were significantly lower after the intervention compared to the control (*p* = 0.031 and 0.041). Epigastric pain, abdominal cramps, early satiety, fullness, and nausea were lower but not significantly after the intervention compared to the control (*p* = 0.65, 0.08, 0.39, 0.70, and 0.54, respectively). The proportion of responders was significantly higher after the intervention compared to the control (50% vs. 16%; *p* = 0.012).	There was no specific funding to support the project. Heidi M. Staudacher was funded by an NHMRC grant (APP1084544) at study conception and throughout data collection.	GJH received unrestricted educational support from Bayer Pty Ltd. and the Falk Foundation.
Patcharatrakul [19] 2021 Thailand	Single‐blind crossover RCT	Patients with GER and nonconstipation IBS (Rome III criteria)	21 (21/21) 85% 18–65/50 ± 13	Low FODMAPs rice noodle	High FODMAPs wheat noodle	1 day (breakfast) Washout: 1 week	NR	NR	Abdominal pain, abdominal burning, bloating, nausea/vomiting, and early satiety severity scores (10 cm VAS)	The early satiety severity score was significantly lower after the intervention compared to the control (*p* < 0.05). Bloating, nausea, abdominal pain, and abdominal burning severity scores were not significantly different between groups.	The Ratchadapiseksompotch Fund, Chulalongkorn University, and a grant from The Gastroenterological Association of Thailand.	None
Patcharatrakul [19] 2021 Thailand	Single‐blind crossover RCT	Patients with GER and nonconstipation IBS (Rome III criteria)	21 (21/21) 85% 18–65/50 ± 13	Low FODMAPs rice noodle	High FODMAPs wheat noodle	1 day (breakfast and lunch) Washout: 1 week	NR	NR	Abdominal pain, abdominal burning, bloating, nausea/vomiting, and early satiety severity scores (10 cm VAS)	Bloating and early satiety severity scores were significantly lower after the intervention compared to the control (*p* < 0.05). Abdominal pain, abdominal burn, and nausea severity scores were not significantly different between groups.	The Ratchadapiseksompotch Fund, Chulalongkorn University, and a grant from The Gastroenterological Association of Thailand.	None
Rivière [34] 2021 France	Single‐blind parallel RCT	Patients with symptomatic PPI‐refractory GERD	31 (16/15) 55% 45 ± 12	LFD	Traditional dietary advice for GER and lifestyle modifications	4 weeks	Yes	Daily food intake diary	RDQ dyspepsia subscore	RDQ dyspepsia subscore was not significantly different between groups.	This study received financial support from the Research Support Fund of the Société Nationale Française de Gastro‐ Entérologie.	PR consultancy fees from AbbVie, Amgen, and Janssen. CM con‐ sultant/speaker for MSD, Kyowa Kirin, Norgine, Biocodex, Mayoly Spindler, Tillots, and Ipsen. SR received research grants from Diversatek Healthcare and Medtronic and was a consultant for Reckitt Benckiser. SB consultant/speaker for MSD, IPSEN, Alfasigma, Abbott, Biocodex, and Mayoli. FM consultant/speaker for Laborie, MSD, Mayoly Spindler, Alfasigma, and Schwa Medico. GG consult‐ ant for Allergan, Takeda, Biocodex, Kyowa Kirin, and Sanofi and is a speaker for Mayoly Spindler, Biocodex, Kyowa Kirin, and Laborie. SS Medtronic. FZ consultant/speaker for Takeda, Allergan, Biocodex, Vifor Pharma, Mayoly Spindler, Ipsen, Abbott, Reckitt Benckiser, and Alfasigma. BV and ER none
Wilson [35] 2020 UK	Double‐blind parallel RCT	Patients with IBS (Rome III criteria)	45 (22/23) 55.5% 18–65/34.69 ± 10.71^c^	LFD advice	Sham dietary advice	4 weeks	NR	Self‐reporter dietary adherence	Abdominal pain, bloating, and nausea severity and incidence scores based on GSRS (4‐point scale)	Abdominal pain, bloating, and nausea severity scores were lower but not significantly after the intervention compared to control (*p* = 0.18, 0.71, and 0.37, respectively). Abdominal pain, bloating, and nausea incidence scores were lower but not significantly after the intervention compared to control (*p* = 0.12, 0.17, and 0.08, respectively)	Clasado Biosciences Ltd. and King's College London.	B.W. was supported by a doctoral research grant from Clasado Biosciences Ltd. M.R. has received funding from the Almond Board of California and the International Nut and Dried Fruit Council. G.C.P. has received personal payments/honoraria/speaker fees from AbbVie, Allergan, Ferring, Janssen, Napp, Takeda, and Tillotts and travel grants or fellowships from AbbVie, Janssen, Takeda, and Tillotts. P.M.I. was a coapplicant of a research grant from Clasado Biosciences Ltd. M.C.L. is the co‐inventor of a mobile app to support patients following the low FODMAP diet and was a co‐applicant of a research grant from Clasado Biosciences Ltd. K.W. has served as a consultant for Danone, has received research funding from Clasado Biosciences, Danone, Nestec Ltd., the Almond Board of California, and the International Nut and Dried Fruit Council, and is the coinventor of a mobile app to support patients following the LFD. T.K., S.A., and A.J.M. have nothing to disclose. The research was performed at King's College London, Guy's and St Thomas’ NHS Foundation Trust, and Barts Health NHS Trust, and stool samples were also analyzed for F.I.S.H at Clasado Biosciences, Reading, UK.
Linlawan [18] 2019 Thailand	Single‐blind crossover RCT	Patients with nonconstipation IBS (Rome III criteria)	20 (20/20) 65% NR/46 ± 11	Low FODMAPs rice noodle	High FODMAPs wheat noodle	1 day (breakfast and lunch) Washout: 1 week	NR	NR	Abdominal pain, abdominal burning, bloating, nausea, and early satiety severity scores (10 cm VAS)	Bloating and early satiety severity scores were significantly lower after the intervention compared to the control (*p* < 0.05). Postprandial nausea, abdominal pain, and abdominal burn severity scores were not significantly different between groups.	The Ratchadapisek Sompotch Endowment Fund (Center of Excellence on Neurogastroenterology and Motility, Chulalongkorn University) and APC were funded by Chulalongkorn University, Bangkok, Thailand.	None
Linlawan [18] 2019 Thailand	Single‐blind crossover RCT	Patients with nonconstipation IBS (Rome III criteria)	20 (20/20) 65% NR/46 ± 11	Low FODMAPs rice noodle	High FODMAPs mung bean noodle	1 day (breakfast and lunch) Washout: 1 week	NR	NR	Abdominal pain, abdominal burning, bloating, nausea, and early satiety severity scores (10 cm VAS)	Abdominal pain, abdominal burning, bloating, nausea/vomiting, and early satiety scores were not significantly different after the intervention compared to control (*p* > 0.05).	The Ratchadapisek Sompotch Endowment Fund (Center of Excellence on Neurogastroenterology and Motility, Chulalongkorn University) and APC were funded by Chulalongkorn University, Bangkok, Thailand.	None
Geysen [36] 2019 Belgium	Single‐blind cross‐over RCT	Healthy individuals	20 (20/20) 50% 32.6 ± 2.8	High‐caloric meal (740 kcal) with 40 g of glucose.	High‐caloric meal (740 kcal) with 40 g of fructose.	1 meal	NR	Direct observation	Bloating and fullness severity scores (VAS NR mm). The proportion of participants with bloating	Bloating and fullness severity scores were not significantly different between groups. The proportion of participants with bloating was not significantly different between groups.	Flemish government, Grant/Award Number: Methusalem grant; Fonds Wetenschappelijk Onderzoek	None
Geysen [36] 2019 Belgium	Single‐blind cross‐over RCT	Healthy individuals	20 (20/20) 50% 32.6 ± 2.8	High‐caloric meal (740 kcal) with 40 g of glucose.	High‐caloric meal (740 kcal) with 40 g of fructans.	1 meal	NR	Direct observation	Bloating and fullness severity scores (VAS NR mm). The proportion of participants with bloating	Bloating and fullness severity scores were not significantly different between groups. The proportion of participants with bloating was not significantly different between groups.	Flemish government, Grant/Award Number: Methusalem grant; Fonds Wetenschappelijk Onderzoek	None
Pirkola [37] 2018 Finland	Double‐blind crossover RCT	Patients with IBS (Rome III criteria)	7 (7/7) 100% 29–51/39^b^	Low‐FODMAPs bread	Regular rye bread	1‐day washout ≥ 2 week	NR	Daily food intake diary	Abdominal pain, abdominal cramps, bloating, nausea, and unpleasant sensation in the upper abdomen severity scores (100 mm VAS)	Abdominal pain, abdominal cramps, bloating, belly rumbling, nausea, and unpleasant sensation in the upper abdomen severity scores were lower but not significantly after the intervention compared to control (*p* = 0.73, 0.91, 0.86, 0.39, and 1.00, respectively).	Fazer Bakeries funded the study and provided the bread.	Laatikainen R has written a Finnish book on irritable bowel syndrome and diet; He is also the founder and owner of Boston Ltd., which provides IBS‐related dietetic services to IBS patients, healthcare professionals, and various organizations; Pirkola L, Hongisto SM, and Loponen J. are employees of Fazer Bakeries; at the time of the research, Pirkola L was working at the University of Helsinki; others have no personal interests to declare;
Skodje [38] 2018 Norway	Double‐blind crossover RCT	Patients with self‐reported NCWS	59 (59/59) 90% 18–80/43.7 ± 12.1	Placebo bar	Fructans bar (2.1 g/day)	1 week	Yes	Daily food intake diary	Pain, bloating, and early satiety dimensions based on GSRS‐IBS (10 cm VAS)	Pain, bloating, and early satiety dimensions scores were lower but not significantly after the intervention compared to control (*p* = 0.07, 0.07, and 0.15, respectively).	This study was funded by the Extra Foundation Health and Rehabilitation, the Norwegian Celiac Association, the Throne Holst Foundation for Nutrition Research, and the Wedel Jarlsberg Foundation.	Peter Gibson has published an information/recipe book on the low FODMAP diet, and his University and Department receive royalties from the sale of The Monash University low FODMAP Diet App. The remaining authors have nothing additional to disclose.
Laatikainen [39] 2017 Finland	Double‐blind parallel RCT	Patients with IBS and self‐reported NCWS (Rome III criteria)	26 (13/13) 96% 21–64/43^b^	Low FODMAPs sourdough bread (fructans 0.06 g/100 g)	High FODMAPs yeast‐fermented bread (fructans 0.23 g/100 g)	1 week	NR	NR	Abdominal pain, abdominal cramps, bloating, nausea, and dyspepsia severity scores (100 mm VAS)	Abdominal pain, abdominal cramps, bloating, nausea, and dyspepsia severity scores were not significantly different between groups (*p* = 0.44, 0.06, 0.93, 0.29, 0.25).	Fazer Bakeries funded the study, provided the bread, and covered the costs of open‐access publishing.	R.L. has written a Finnish book on irritable bowel syndrome and diet. He is also a founder and owner of Boston Oy Ltd. (Helsinki, Finland), which provides IBS‐related dietetic services to IBS patients, health care professionals, and various organizations. S.‐M.H. and J.L. are employees of the Fazer Group (Helsinki, Finland). T.P. has received consultation fees from Boston Ltd. The other authors have no personal interests to declare.
Staudacher [40] 2017 UK	Single‐blind crossover RCT	Patients with IBS (Rome III criteria)	104 (51/53) 67% 18–65/35.49 ± 11.78	LFD advice	Sham dietary advice	4 weeks	Yes	Daily food intake diary	Abdominal pain, bloating, and nausea severity scores based on GSRS (4‐point scale)	Abdominal pain and bloating severity scores were significantly lower after the intervention compared to the control (*p* = 0.01 and 0.001). Nausea severity score was not significantly different between diets (*p* 0.53).	The National Institute for Health Research funded the study. FMF and PL receive financial support from the Scottish Government Rural and Environmental Sciences and Analytical Services (RESAS).	None
Laatikainen [41] 2016 Finland	Double‐blind crossover RCT	Patients with IBS (Rome III criteria)	87 (87/87) 91.3% 21–64/42.9^b^	Low FODMAPs rye bread (fructan 0.3 g and mannitol 0.1 g)	Regular rye bread	4 weeks washout ≥ 4 weeks	Yes	Food record and tick‐box diary	Abdominal pain and dyspepsia severity scores (100 mm VAS)	The abdominal pain severity score was significantly lower after the intervention compared to the control (*p* = 0.049 and 0.001, respectively). The dyspepsia severity score was lower but not significantly after the intervention compared to the control (*p* = 0.06).	Fazer Bakeries funded the study and provided the bread.	Reijo Laatikainen has written a Finnish book on irritable bowel syndrome and diet. He is also the founder and owner of Boston Ltd., which provides IBS‐related dietetic ser‐ vices to IBS patients, healthcare professionals, and various organizations. Sanna‐Maria Hongisto and Jussi Loponen are employees of Fazer Bakeries. Tuija Poussa has received consultation fees from Boston Ltd. Others have no personal interests to declare.
Staudacher [42] 2012 UK	Parallel RCT	Patients with IBS (Rome III criteria)	35 (16/19) 65.7% 18–65/35.91 ± 10.17^c^	LFD advice	Habitual diet	4 weeks	Yes	Daily food intake diary	Abdominal pain, bloating, and nausea incidence and severity scores (4‐point scale)	Abdominal pain and bloating incidence and severity scores were significantly lower after the intervention compared to the control (Incidence: *p* = 0.002 and 0.02 ‐ Severity: *p* = 0.002 and 0.07) Nausea incidence and severity scores were lower but not significantly after the intervention compared to the control (Incidence: *p* = 0.67 ‐ Severity: *p* = 0.64)	The British Dietetic Association Research Award and Guy's and St. Thomas’ Charity (H.M.S.)	None
Ong [43]2010 Australia	Single‐blind crossover RCT	Patients with IBS (Rome III criteria)	15 (15/15) 86% 22–59/41^b^	Provided foods for LFD (FODMAPs: 9 g/day)	Provided foods for high FODMAPs diet (FODMAPs: 50 g/day)	2 days Washout 7 days	NR	Daily food intake diary	Abdominal pain/discomfort, bloating, and nausea severity scores (4‐point scale)	Abdominal pain/discomfort, bloating, and nausea severity scores were significantly lower after the intervention compared to the control (*p* = 0.006, 0.002, and 0.01, respectively)	This work was supported by the National Health and Medical Research Council (NHMRC) of Australia and the Vera and Les Erdi Foundation.	S.J.S. has published cookbooks directed toward issues of dietary fructan restrictions, fructose malabsorption, and celiac disease. She has also published shopping guides for low FODMAPs and low fructose and fructan foods.
Ong [43] 2010 Australia	Single‐blind crossover RCT	Healthy individuals	15 (15/15) 60% 22–68/23^b^	Provided foods for LFD (FODMAPs: 9 g/day)	Provided foods for high FODMAPs diet (FODMAPs: 50 g/day)	2 days Washout 7 days	NR	Daily food intake diary	Abdominal pain/discomfort, bloating, and nausea severity scores (4‐point scale)	Abdominal pain/discomfort, bloating, and nausea severity scores were not significantly different between groups (*p* = 0.145, 0.484, and 0.773, respectively).	This work was supported by the National Health and Medical Research Council (NHMRC) of Australia and the Vera and Les Erdi Foundation.	S.J.S. has published cookbooks directed toward issues of dietary fructan restrictions, fructose malabsorption, and celiac disease. She has also published shopping guides for low FODMAPs and low fructose and fructan foods.

Abbreviations: FD, functional dyspepsia; FODMAP, fermentable oligo‐, di‐ and monosaccharides and polyols; GER, gastroesophageal reflux; GFD, gluten‐free diet; GSRS‐IBS, gastrointestinal symptom rating score irritable bowel syndrome questionnaire; GSRS, gastrointestinal symptom rating score questionnaire; IBS, irritable bowel syndrome; LFD, low‐FODMAP diet; NCWS, nonceliac wheat sensitivity; NICE, National Institute for Clinical Excellence; NR, not reported; RCT, randomized controlled trial; RDQ, Reflux Disease Questionnaire; SAGIS, Structured Assessment of Gastrointestinal Symptoms questionnaire; SF‐NDI, short‐form Nepean Dyspepsia Index questionnaire; TDA, traditional dietary advice; VAS, visual analog scale.

^a^ The data are represented as the range/mean ±SD unless indicated otherwise.

^b^ The data are presented as the combined means ±SD.

^c^ The data are represented as range/median values.

^d^ Numbers are retrieved from per‐protocol data.

Dyspeptic symptoms were assessed using various questionnaires: the Nepean Dyspepsia Index (NDI) symptom checklist [15], Structured Assessment of Gastrointestinal Symptoms (SAGIS) [17], GSRS [35, 40], GSRS‐IBS [38], and Reflux Disease Questionnaire (RDQ) [34]. Researcher‐designed surveys focused on abdominal pain, bloating, and nausea as the only dyspeptic symptoms [42, 43], or along with early satiety [16, 18, 19], abdominal burning [18, 19], abdominal cramps [37, 39], or fullness [36]. Culturally adapted measures included the term dyspepsia as a symptom (adopted from Finnish, indicating an unpleasant sensation in the upper abdomen) [37, 39, 41].

The characteristics of the 18 included NRSIs (24 arms) are summarized in Table 2. Studies were conducted on patients with FD and fructose or lactose intolerance [56], FD and self‐reported NCWS [50], FGIDs including FD [55], IBS [44, 45, 46, 47, 48, 51, 53, 54, 57, 58, 59, 60, 61], chronic diarrhea [49], self‐reported NCWS [52], and healthy individuals [52]. The interventions of most studies involved LFD advice from a dietician [44, 45, 46, 47, 48, 49, 50, 51, 52, 53, 56, 57, 58, 59, 60, 61], except for two [54, 55]. In one, participants initially followed a 3‐day LFD and later consumed researcher‐provided foods for a high galacto‐oligosaccharide diet, a specific type of FODMAPs [54]. In the other, fructose (35 g) or lactose (50 g) dissolved in water was prescribed before the breath test [55]. The intervention duration varied from 5 h [55] to 12 months [53]. Dyspeptic symptoms were evaluated using the NDI symptom checklist [50], SAGIS [48, 49], GSRS [52, 58], or questionnaires including abdominal pain, bloating, and nausea as the only dyspeptic symptoms [44, 45, 51, 53, 54, 57, 59, 60] or along with fullness [46, 55, 61], postprandial discomfort [46], or idiopathic abdominal pain [47]. One study merely reported the proportion of FD patients who achieved adequate relief [56].

**Table 2:** Characteristics of NRSIs included in the systematic review.

First author Year Location	Study design	Population (diagnostic criteria)	Number (intervention/comparison) female (%) age^a^ (y)	Intervention	Duration (week, day, hour)	Dietitian involvement	Adherence monitoring	Outcome (outcome measurement tool)	Result	Source of funding	Conflict of interest
Valdez‐Palomares [44] 2021 Mexico	NRSI	Patients with IBS (Rome III criteria)	32 90% 18–60/NR	LFD advice	4 weeks	Yes	Daily food intake diary	Abdominal pain, flatulence/bloating, nausea/vomiting severity scores (100 mm VAS)	In the responders’ group (improvement in the main IBS symptom), abdominal pain, bloating, and nausea severity scores were significantly lower after intervention (*p* < 0.0001, < 0.0001, and < 0.05, respectively). In the nonresponders’ group, only the bloating severity score was significantly lower after intervention (*p* < 0.05).	NR	None
Seamark [45] 2021 UK	Observational service evaluation	Patients with IBS (Rome IV criteria)	547 NR NR	LFD advice	9 (9*–*13)^d^ weeks	Yes	NR	The proportion of patients with moderate or severe abdominal pain/discomfort, abdominal bloating/distension, and nausea.	The proportion of patients with moderate or severe abdominal pain/discomfort, abdominal bloating/distension, and nausea was significantly lower after intervention (*p* < 0.001).	British Dietetic Association, Grant/Award Number: General Education Trust Funding	None
Seamark [45] 2021 UK	Observational service evaluation	Patients with IBS (Rome IV criteria)	211 86% NR/53.6 ± 15	LFD advice	13 (12– 16)^d^ months	Yes	NR	The proportion of patients with moderate/severe abdominal pain/discomfort, abdominal bloating/distension, and nausea.	The proportion of patients with moderate/severe abdominal pain/discomfort, abdominal bloating/distension, and nausea was significantly lower after the intervention (*p* < 0.01).	British Dietetic Association, Grant/Award Number: General Education Trust Funding	None
Yang [46] 2021 Singapore	Prospective trial	Patients with IBS (criteria: clinicians’ opinion)	50 72% NR/47.3 ± 16.9	LFD advice	3–6 weeks	Yes	Dietitian judgment	Abdominal pain/discomfort, postprandial pain/discomfort, bloating, nausea, and fullness severity scores (10 cm VAS)	Abdominal pain/discomfort, postprandial pain/discomfort, bloating, nausea, and fullness severity scores were significantly lower after the intervention (*p* < 0.01).	None	None
Ostrowska [47] 2021 Switzerland	Open‐label trial (control data considered as a single arm trial)	Patients with mixed IBS (Rome III criteria)	52 (26/26) 100% 42.2 ± 14.99^b^	LFD advice	8 weeks	Yes	NR	The proportion of patients with postprandial abdominal pain, bloating, nausea idiopathic abdominal pain, and gastric fullness (Yes/no question)	The proportion of patients with postprandial abdominal pain, bloating, and gastric fullness was significantly lower after the intervention. The proportion of patients with complete resolution in idiopathic abdominal pain and nausea was not significantly different after the intervention.	The study was funded by internal sources of the Medical University of Bialystok (Project No.: R‐I‐002/389/2015).	None
Chan [48] 2020 New Zealand	Feasibility study	Patients with IBS (Rome IV criteria)	11 NR 34.5^c^	LFD advice	6 weeks	Yes	Dietitian judgment	Epigastric pain, postprandial pain, abdominal cramps, bloating, nausea, fullness, and early satiety severity scores based on SAGIS (5‐point scale)	Epigastric pain, postprandial pain, abdominal cramps, bloating, nausea, and fullness severity scores were significantly lower after the intervention (*p* = 0.01, 0.000, 0.01, 0.01, 0.02, and 0.01, respectively). Early satiety severity score was lower but not significantly after the intervention (*p* = 0.17).	NR	None
O'Brien [49] 2020 New Zealand	Non‐blinded intervention study	Adults older than 65 years with chronic diarrhea and normal colonoscopy	20 75% 67–84/76^c^	LFD advice	6 weeks	Yes	Daily food intake diary	Epigastric pain/discomfort and Nausea/vomiting domains based on SAGIS (4‐point scale)	The nausea/vomiting domain score was lower but not significantly after the intervention (*p* = 0.214). Epigastric pain/discomfort domain score was significantly lower after the intervention (*p* < 0.005).	None	None
Potter [50] 2020 Australia	NRSI (the phase before enrolling RCT)	Patients with FD (Rome III criteria)	9 75% 8–80/NR	LFD and GFD advice	4 weeks	Yes	Food frequency questionnaire	Total severity score based on NDI symptom checklist (10 cm VAS)	Total severity score was lower but not significantly after the intervention (*p* = 0.087)	A Ph.D. scholarship grant to MP from the National Health andMedical Research Council and a National Health & Medical Research Council Investigator Grant (NJT)	None
Frieling [51] 2019 Germany	Prospective study	Patients with IBS (Rome III criteria)	33 63.6% 42 ± 19^b^	LFD advice	8 weeks	Yes	NR	Abdominal pain, flatulence/bloating, and nausea/vomiting severity scores (3‐point scale)	In the responders’ group (improvement in the main IBS symptom), abdominal pain, flatulence/bloating, and nausea/vomiting severity scores were significantly lower after the intervention. In the nonresponders’ group, all symptom severity scores were not significantly lower after the intervention.	NR	None
Dieterich [52] 2019 USA	NRSI	Patients with Self‐reported NCWS	19 79% 33.8 ± 11.9	LFD advice	2 weeks	Yes	Daily food intake diary	Abdominal pain dimension score based on GSRS (10 cm VAS)	Abdominal pain dimension score was significantly lower after the intervention (*p* < 0.01).	J. & F. Marohn‐Stiftung, University of Erlangen‐Nürnberg, Germany, Dr. Sch€ar AG/SPA, Burgstall Italy, Leibniz‐Gemeinschaft Germany (WGL, project SAW‐2016‐DFA‐2), and H.W. & J. Hector Stiftung, Weinheim Germany.	None
Dieterich [52] 2019 USA	NRSI	Healthy individuals	10 70% 32.8 ± 10.9	LFD advice	2 weeks	Yes	Daily food intake diary	Abdominal pain dimension score based on GSRS (10 cm VAS)	The abdominal pain dimension score was not significantly different after the intervention (*p* = 0.332).	J. & F. Marohn‐Stiftung, University of Erlangen‐Nürnberg, Germany, Dr. Sch€ar AG/SPA, Burgstall Italy, Leibniz‐Gemeinschaft Germany (WGL, project SAW‐2016‐DFA‐2), and H.W. & J. Hector Stiftung, Weinheim Germany.	None
O'Keeffe [53] 2018 UK	Prospective long‐term follow‐up postal questionnaire study	Patients with IBS (NICE criteria)	103 74% 49 ± 15	LFD advice	6 weeks	Yes	Food frequency questionnaire	The proportion of patients with abdominal pain, bloating, and nausea	The proportion of patients with abdominal pain and bloating was significantly lower after the intervention (all *p* < 0.001). The proportion of patients with nausea was lower but not significantly after the intervention (*p*=NR).	Dr Schär; part‐ funding.	None
O'Keeffe [53] 2018 UK	Prospective long‐term follow‐up postal questionnaire study.	Patients with IBS (NICE criteria)	103 74% 49 ± 15	LFD advice	12 months	Yes	Food frequency questionnaire	The proportion of patients with abdominal pain, bloating, and nausea	The proportions of patients with abdominal pain and bloating were significantly lower after the intervention (*p* < 0.001 and 0.001). The proportion of patients with nausea was lower but not significantly after the intervention (*p*=NR).	NR	None
Tuck [54] 2018 Australia	Control data considered as a single arm trial	Patients with IBS (Rome III criteria)	31 90.3% 21–64/34^c^	Provided foods for a high Galacto‐Oligosaccharides diet	3 days	Yes	Daily food intake diary	Abdominal pain, bloating, and nausea severity scores (100 mm VAS)	Abdominal pain, bloating, and nausea severity scores were significantly higher after the intervention (*p* = 0.003, 0.0001, and 0.003, respectively).	The Australian National Health and Medical Research Council (NHMRC)	None
Wilder‐Smith [55] 2018 Switzerland	NRSI by clinical practice data	Patients with FGIDs including FD (Rome III criteria)	2042 72% > 18/41.1 ± 16.6	Fructose (35 g) during breath test	5 h	NR	NR	The proportion of patients with mild/severe abdominal pain, bloating, nausea, and fullness (3‐point scale)	The proportion of patients with mild/severe fullness or bloating increased 2 h after the intervention and then decreased over the next 3 h.	NR	None
Wilder‐Smith [55] 2018 Switzerland	NRSI by clinical practice data	Patients with FGIDs including FD (Rome III criteria)	2042 72% > 18/41.1 ± 16.6	Lactose (50 g) during breath test	5 h	NR	NR	The proportion of patients reporting mild or severe abdominal pain, bloating, nausea, and fullness (3‐point scale)	The proportion of patients reporting mild or severe fullness or bloating increased 2 h after the intervention and then decreased over the next 3 h.	NR	None
Wilder‐Smith [56] 2017 Switzerland	Longitudinal observational study	Patients with FD and fructose or lactose intolerance (Rome III criteria)	193 NR NR	LFD advice	4 weeks	Yes	Interview	The proportion of patients achieving global adequate relief (yes/no question)	77% of patients achieved global adequate relief.	Guarantor of the article: Clive Wilder‐Smith	None
Peters [57] 2016 Australia	Control data considered as a single arm trial	Patients with IBS (Rome III criteria)	24 79% 23–66/34^c^	LFD advice	6 weeks	Yes	Interview	Abdominal pain, bloating, and nausea severity scores (100 mm VAS)	Abdominal pain, bloating, and nausea severity scores were significantly lower after the intervention (*p* < 0.0001).	The Department of Gastroenterology, Monash University. Simone L.	None
Peters [57] 2016 Australia	Control data considered as a single arm trial	Patients with IBS (Rome III criteria)	24 79% 23–66/34^c^	LFD advice	6 months	Yes	Interview	Abdominal pain, bloating, and nausea severity scores (100 mm VAS)	Abdominal pain, bloating, and nausea severity scores were significantly lower after the intervention (*p* < 0.0001).	The Department of Gastroenterology, Monash University. Simone L.	None
Whigham [58] 2015 UK	NRSI	Patients with IBS (NICE criteria)	263 70% 17–81/40^c^	LFD advice: group education	11.3 ± 10^e^ weeks	Yes	Interview	The proportion of patients with moderate/severe abdominal pain, bloating, and nausea based on GSRS (4‐point scale)	The proportion of patients with moderate/severe abdominal pain, bloating, and nausea was significantly lower after the intervention (*p* < 0.001).	Lucy Whigham was funded by a clinical academic training program internship grant from NHS London	None
Whigham [58] 2015 UK	NRSI	Patients with IBS (NICE criteria)	101 66% 18–91/46^c^	LFD advice: one‐to‐one education	11.3 ± 10^e^ weeks	Yes	Interview	The proportion of patients with moderate/severe abdominal pain, bloating, and nausea based on GSRS (4‐point scale)	The proportion of patients with moderate/severe abdominal pain, bloating, and nausea was significantly lower after the intervention (*p* < 0.001, < 0.001, and 0.002, respectively).	Lucy Whigham was funded by a clinical academic training program internship grant from NHS London	None
Huamán [59] 2015 Spain	NRSI	Patients with IBS and functional bloating (Rome III criteria)	30 80% > 18/39 ± 12	LFD advice	2 months	Yes	Questionnaire	The proportion of patients with at least 5 point reduction in abdominal pain, bloating, and nausea (10 cm VAS)	83%, 73%, and 76% of patients reported at least 5 point reduction in abdominal pain, bloating, and nausea after the intervention.	None	None
Biesiekierski [60] 2013 Australia	Control data considered as a single arm trial	Patients with IBS and Self‐reported NCWS (Rome III criteria)	37 83.7% 24–61/45^c^	LFD advice	2 weeks	NR	Tick‐box diary, unused food count, Daily food intake diary	Abdominal pain, bloating, and nausea severity scores (100 mm VAS)	Abdominal pain and bloating severity scores were significantly lower after the intervention (*p* <0.0001). Nausea severity score was not significantly different after the intervention (*p* = 0.149).	George Weston Foods as part of a partnership in an Australian Research Council Linkage Project and the National Health and Medical Research Council (NHMRC) of Australia.	None
De Roest [61] 2013 New Zealand	Prospective observational study	Patients with IBS (criteria: NR)	90 84.4% NR/47.0 ± 15.3	LFD advice	15.7 ± 9.0^e^ months	Yes	Questionnaire	Abdominal pain, bloating, nausea, fullness shortly after eating, and fullness even long after eating severity scores (7‐point scale)	Abdominal pain, bloating, nausea, and fullness shortly after eating severity scores were significantly lower after the intervention (*p* < 0.0001, 0.001, respectively) However, fullness even long after eating severity score was not significantly lower after intervention (*p* = 0.051)	NR	None

Abbreviations: FD, functional dyspepsia; FGIDs, functional gastrointestinal disorders; GFD, gluten‐free diet; GSRS, Gastrointestinal Symptom Rating Score questionnaire; IBS, irritable bowel syndrome; LFD, low‐FODMAP diet; NCWS, nonceliac wheat sensitivity; NDI, Nepean Dyspepsia Index questionnaire [15]; NICE, National Institute for Clinical Excellence; NR, not reported; NRSI, non‐ randomized studies of interventions; SAGIS, Structured Assessment of Gastrointestinal Symptoms questionnaire; VAS, visual analog scale.

^a^ The data are represented as the range/mean ±standard deviation unless indicated otherwise.

^b^ The data are presented as combined means ±and standard deviations.

^c^ The data are represented as range/median values.

^d^ The data are represented as median (range) values.

^e^ The data are represented as the mean ±standard deviation.

### 3.3 Risk of Bias

*In RCTs,* one study had high selection bias [17], while the others had a low risk [15, 16, 18, 19, 34, 35, 36, 37, 38, 39, 40, 41, 42, 43]. In terms of performance bias, almost two‐thirds of the studies had a high risk of bias [15, 16, 17, 18, 19, 34, 35, 36, 40, 43], whereas five had a low risk of bias [37, 38, 39, 41, 42]. Detection bias was low in all studies except for two, which had a high risk [17]. In the domain of attrition bias, two studies raised some concerns [16, 17] and the rest had a low risk of bias [15, 18, 19, 34, 35, 36, 37, 38, 39, 40, 41, 42, 43]. Regarding reporting and other biases, all studies were deemed to have a low bias (Figure 2a).

Figure 2(a) The methodological quality of the included randomized controlled trial (RCT). (b) The methodological quality of the included non‐randomized studies of interventions (NRSI).

**Figure:**
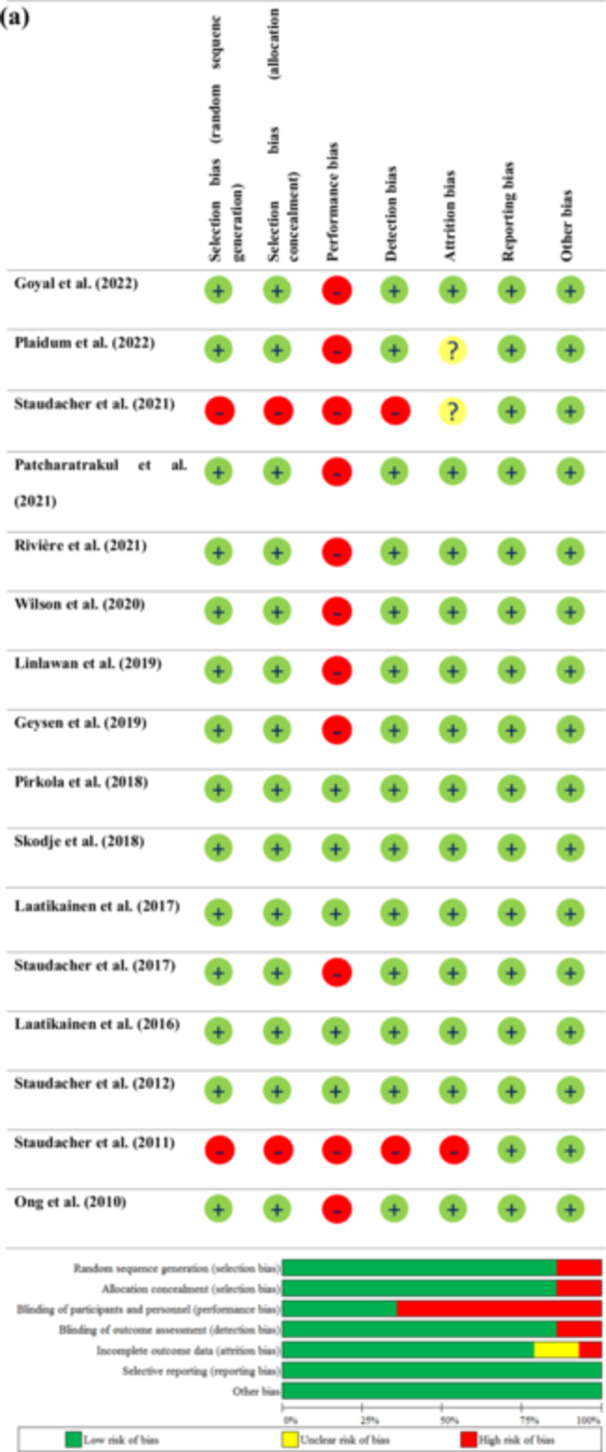

**Figure:**
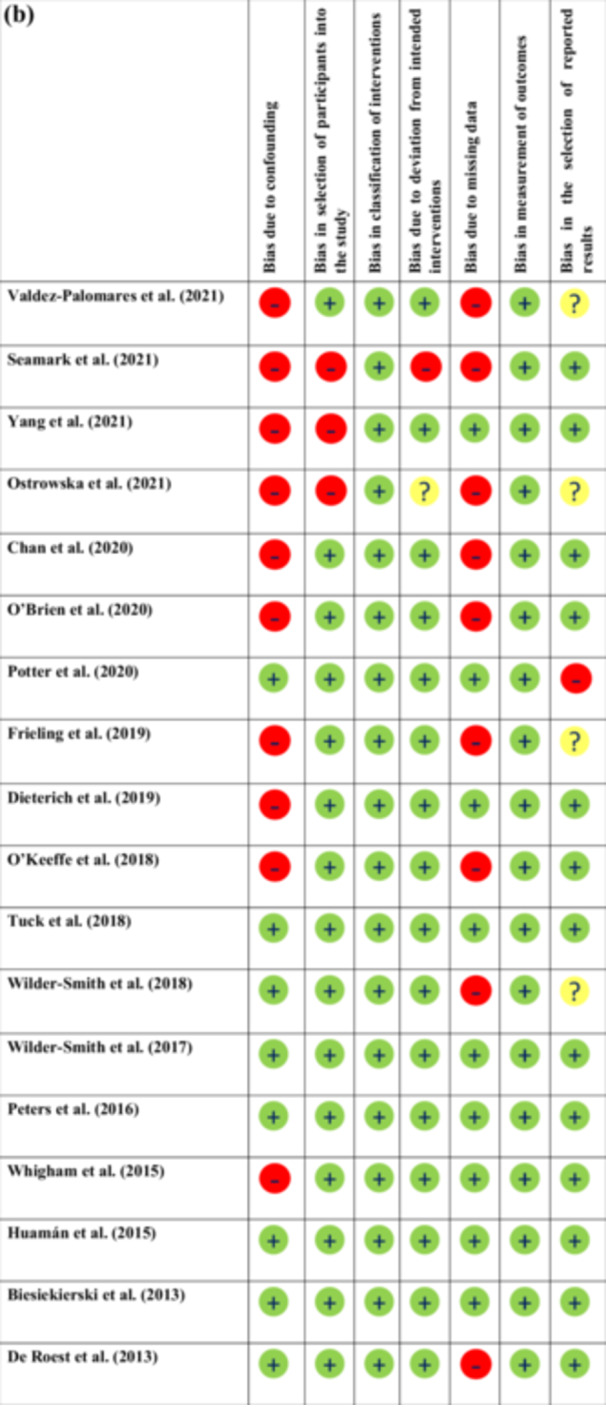

*In NRSIs*, all exhibited a moderate to serious risk of bias. Specifically, two‐thirds had a high risk of confounding bias [44, 45, 46, 47, 48, 49, 51, 52, 53, 58], while the remaining had a low risk [50, 54, 55, 56, 57, 59, 60, 61]. Three studies had a high risk of bias in the selection of participants [45, 46, 47], but the remaining studies had a low risk [44, 48, 49, 50, 51, 52, 53, 54, 55, 56, 57, 58, 59, 60, 61]. In the sections “Classification of Intervention” and “Outcome Measurement,” all studies exhibited a low risk of bias. In “deviation from intended interventions,” one study had a high risk [45], and one had some concerns [47], but the rest had a low risk of bias [44, 46, 48, 49, 50, 51, 52, 53, 54, 55, 56, 57, 58, 59, 60, 61]. Bias due to missing data was high in half of the studies [44, 45, 47, 48, 49, 51, 53, 55, 61], but low in the others [46, 50, 52, 54, 56, 57, 58, 59, 60]. Most studies had a low risk in “selection of reported results” [45, 46, 48, 49, 52, 53, 54, 56, 57, 58, 59, 60, 61]; however, high and moderate risk were observed in one [50] and four [44, 47, 51, 55] studies, respectively (Figure 2b).

### 3.4 Findings From Systematic Review Conducted via RCTs

*In patients*, the severity of abdominal pain was evaluated in 13 studies (17 intervention arms). A significant reduction in the LFD compared to that of controls was demonstrated in five studies involving 206 patients [17, 40, 41, 42, 43], while the remaining articles (401 patients) did not show improvement [15, 16, 18, 19, 35, 37, 38, 39, 41]. Among the 12 RCTs examining bloating severity (16 arms), nine RCTs including 238 subjects reported a significant reduction in the LFD [15, 16, 17, 18, 19, 39, 40, 42, 43], whereas other studies with 157 subjects showed no difference [18, 19, 35, 37, 39]. When examining nausea severity across 12 studies (15 arms), LFD reduced nausea in only one study involving 15 individuals [43], with the remaining RCTs (508 individuals) failing to reveal significant changes [15, 16, 17, 18, 19, 35, 37, 38, 39, 40, 41, 42, 43]. Early satiety was observed in nine intervention arms from six studies [15, 16, 17, 18, 19, 38], with a significant reduction noted in most cases (178 patients) [15, 16, 18, 19], except for three arms (69 patients) [17, 18, 38]. Regarding fullness severity, two studies (three arms) involving 108 patients reported a significant reduction in the LFD in two groups [15] but not in one group with 40 patients [17]. In contrast, LFD did not significantly alter the severity of epigastric cramps in 168 patients [15, 17, 37, 39], abdominal burning in 168 patients [15, 17, 18], dyspepsia in 107 patients [37, 39, 41], epigastric discomfort in 108 patients [15], or the RDQ dyspepsia subscore in 16 patients [34]. In one study, an LFD reduced the frequency of abdominal pain and bloating [42], while another study found no significant effect on these symptoms [35]. Neither study reported any impact on nausea frequency [35, 42] (Table 3).

**Table 3:** Effects of various intervention types (low‐FODMAP diet and FODMAP challenge) on the severity of symptoms in RCTs.

	Number of studies	Number of intervention arms	The number of intervention arms showing a significant change	The number of intervention arms not showing a significant change
Abdominal pain	13	17	Five [17, 40, 41, 42, 43]	Twelve [15, 16, 18, 19, 35, 37, 38, 39, 41, 43]
Fullness/bloating	12	16	Nine [15, 16, 17, 18, 19, 39, 40, 42, 43]	Seven [18, 19, 35, 37, 38, 39, 43]
Nausea	12	15	One [43]	Fourteen [15, 16, 17, 18, 19, 35, 37, 38, 39, 40, 41, 42, 43]
Early satiety	6	9	Six [15, 16, 18, 19]	Three [17, 18, 38]
Epigastric cramps	4	5	Zero	Five [15, 17, 37, 39]
Epigastric burning	3	5	Zero	Five [15, 17, 18]
Epigastric discomfort (dyspepsia)	5	6	Zero	Six [15, 34, 37, 39, 41]

*In healthy subjects*, two RCTs reported no significant reduction in the severity of abdominal pain, bloating, and nausea [43] or bloating and fullness [36]. Similarly, the proportion of participants with bloating did not significantly differ between the LFD group and the HFD group [36].

### 3.5 Findings From Systematic Review Conducted via NRSIs

*In patients*, the severity of abdominal pain, bloating, and nausea was reported in eight studies (with nine intervention arms and 332 individuals) [44, 46, 48, 51, 54, 57, 60, 61]. All scores were significantly lower after LFD than at baseline, except for the nausea score in one study [60]. The effect of LFD on other symptoms has been poorly examined. Adherence to the LFD could significantly reduce fullness in 151 patients [46, 48, 61], abdominal cramps in 11 patients [48], postprandial pain in 11 patients [48], pain domain of GSRS (including pain, bloating, and nausea) in 19 patients [52], and epigastric discomfort domain (including postprandial pain, epigastric pain, bloating, fullness, early satiety, retrosternal discomfort, and abdominal cramps) in 20 patients [49] but failed to improve the severity of early satiety in 11 patients [48], fullness even long after eating in 90 patients [61], total score of NDI in 9 patients [50], and nausea domain (including loss of appetite, sickness, nausea, and vomiting) in 20 patients [49] (Table 4).

**Table 4:** Effects of various intervention types (low‐FODMAP diet and FODMAP challenge) on the severity of symptoms in NRSIs.

	Number of studies	Number of intervention arms	The number of intervention arms showing a significant change	The number of intervention arms not showing a significant change
Epigastric pain	8	9	Nine [44, 46, 48, 51, 54, 57, 60, 61]	Zero
Fullness/bloating	8	9	Nine [44, 46, 48, 51, 54, 57, 60, 61]	Zero
Nausea	8	9	Eight [44, 46, 48, 51, 54, 57, 61]	One [60]
Early satiety	1	1	Zero	One [48]
Abdominal cramps	1	1	One [48]	Zero

Seven studies (with 11 intervention arms) reported the proportion of responders to an LFD [45, 47, 53, 55, 56, 58, 59]. There was a significant reduction in the proportion of patients with abdominal pain [45, 47, 53, 58], bloating [45, 47, 53, 55, 58], nausea [45, 58], and fullness [47, 55]. However, there was no significant reduction in nausea reported in one study [53] or idiopathic abdominal pain in another study [47]. Specifically, after the intervention, a decrease of at least five points in abdominal pain, bloating, and nausea was observed in 83%, 73%, and 76% of patients, respectively [59], while in another study, 77% of patients experienced adequate global relief [56].

In healthy subjects, one study reported no changes in the pain domain of the GSRS (including pain, bloating, and nausea) in 10 patients after the intervention [52].

### 3.6 Findings From Meta‐Analysis Conducted via RCTs

**Figure 3:**
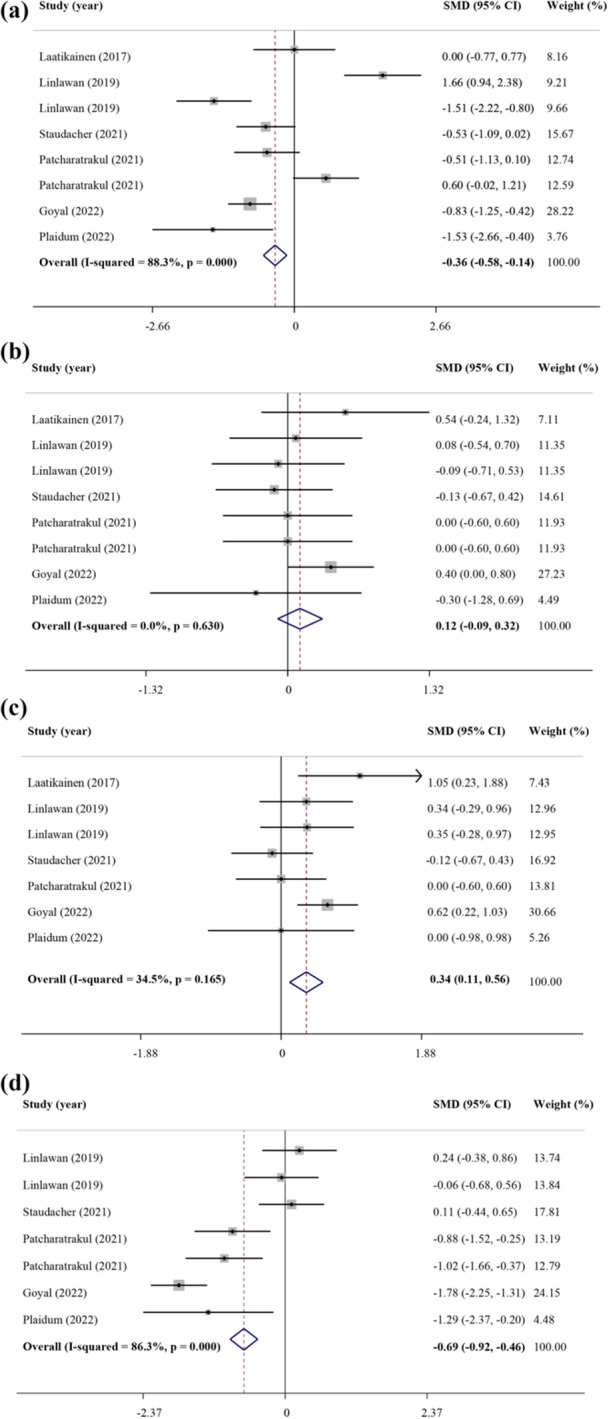
(a) The intervention arm of fermentable oligo‐, di‐ and monosaccharides and polyols (FODMAP) intervention arm in a randomized controlled trial (RCT) on the severity of bloating. (b) The intervention arm of fermentable oligo‐, di‐ and monosaccharides and polyols (FODMAP) intervention arm in a randomized controlled trial (RCT) on the severity of nausea. (c) The intervention arm of fermentable oligo‐, di‐ and monosaccharides and polyols (FODMAP) intervention arm in a randomized controlled trial (RCT) on the severity of pain. (d) The intervention arm of fermentable oligo‐, di‐ and monosaccharides and polyols (FODMAP) intervention arm in a randomized controlled trial (RCT) on the severity of early satiety.

Bloating severity decreased significantly in the LFD group compared to the control group in six RCTs with eight arms (WMD = −0.36, 95% CI: −0.58, −0.14; I2 = 88.3%) (Figure 3a). However, a sensitivity analysis excluding the Goyal et al. study [15] removed the significance, indicating its notable influence. No publication bias was found (Egger's test, *p* = 0.725; Begg's test, *p* = 0.536). As shown in Figure 3b, compared with the control diet, the LFD did not significantly improve nausea severity in the same RCTs. A small positive effect was observed, but it was not substantial (WMD = 0.12, 95% CI: −0.09, 0.32; *I* ^2^ = 0.0%). There was no evidence of publication bias (Egger's test, *p* = 0.241; Begg's test, *p* = 0.805). LFDs were found to increase pain significantly compared to controls in the same RCTs (WMD = 0.34, 95% CI: 0.11, 0.56; *I* ^2^ = 34.5%) (Figure 3c), with no evidence of publication bias (Egger's test, *p* = 0.750; Begg's test, *p* = 0.548). Excluding the study by Goyal et al. removed the significance (WMD = 0.21, 95% CI: −0.06, 0.48). Figure 3d shows the effect of LFD on early satiety. The analysis of the five RCTs with seven arms revealed that compared with the control, the LFD decreased early satiety significantly (WMD = −0.69, 95% CI: −0.92, −0.46; *I* ^2^ = 86.3%). No publication bias was found (Egger's test, *p* = 0.736; Begg's test, *p* = 0.548).

**Figure 4:**
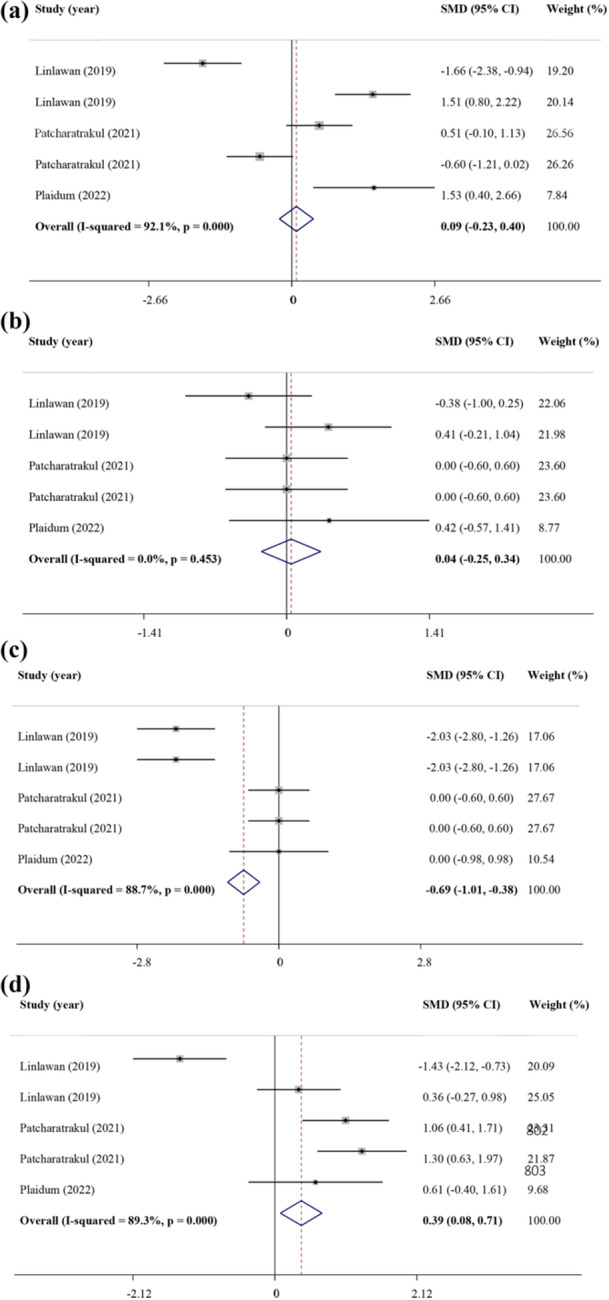
(a) The intervention arm of fermentable oligo‐, di‐ and monosaccharides and polyols (FODMAP) in challenge studies on the severity of bloating. (b) The intervention arm of fermentable oligo‐, di‐ and monosaccharides and polyols (FODMAP) in challenge studies on the severity of nausea. (c) The intervention arm of fermentable oligo‐, di‐ and monosaccharides and polyols (FODMAP) in challenge studies on the severity of pain. (d) The intervention arm of fermentable oligo‐, di‐ and monosaccharides and polyols (FODMAP) in challenge studies on the severity of early satiety.

Additional analyses were conducted on studies involving FODMAPs challenges. These analyses indicated no significant change in bloating (WMD = 0.09, 95% CI: −0.23, 0.40; *I* ^2^ = 92.1%) (Figure 4a). Sensitivity analysis revealed a notable increase after excluding the study by Linlawan et al. [18]. No publication bias was detected (Egger's test, *p* = 0.616; Begg's test, *p* = 1.00). An HFD had no significant impact on nausea (*n* = 6, WMD = 0.04, 95% CI: −0.25, 0.34; *I* ^2^ = 0.0%) (Figure 4b). The sensitivity analysis consistently supported these findings, with no signs of publication bias (Egger's test, *p* = 0.410; Begg's test, *p* = 0.624). FODMAP challenge significantly lowered pain scores compared to the LFD (*n* = 6, WMD = −0.69, 95% CI: −1.01, −0.38; *I* ^2^ = 88.7%) (Figure 4c). Sensitivity analysis confirmed that the exclusion of any single study did not substantially alter the results. No publication bias was evident (Egger's test, *p* = 0.494; Begg's test, *p* = 0.463). The FODMAP challenge increased early satiety significantly (WMD = 0.39, 95% CI: 0.08, 0.71; *I* ^2^ = 89.3%) (Figure 4d). Sensitivity analysis indicated that the exclusion of the study by Patcharatrakul et al. nullified this significance. No publication bias was evident (Egger's test, *p* = 0.920; Begg's test, *p* = 1.00).

### 3.7 Findings From Meta‐Analysis Conducted via NRSIs

Five studies with six arms showed that the LFD significantly decreased postintervention bloating compared to preintervention values (WMD = −2.06, 95% CI: −4.08, −0.05; *I* ^2^ = 99.1%) (Figure S1). No publication bias was detected (Egger's test, *p* = 0.949; Begg's test, *p* = 0.851). Six studies with seven arms indicated a significant decrease in nausea post‐LFD (WMD = −1.15, 95% CI: −2.13, −0.17; *I* ^2^ = 99.4%) (Figure S2). No publication bias was detected (Egger's test, *p* = 0.141; Begg's test, *p* = 1.00). The LFD did not significantly reduce pain severity across the seven arms (WMD = −1.04, 95% CI: −2.26, 0.18; *I* ^2^ = 98.8%) (Figure S3), with no publication bias (Egger's test, *p* = 0.918; Begg's test, *p* = 1.00). Three studies showed that the LFD reduced fullness significantly (WMD = −1.63, 95% CI: −2.92, −0.35; *I* ^2^ = 98.4%). No publication bias was detected (Egger's test, *p* = 0.610; Begg's test, *p* = 1.00) (Figure S4). In all four meta‐analyses, sensitivity analysis revealed that excluding any study did not significantly impact the results.

### 3.8 Quality Assessment

According to the AMSTAR 2, this systematic review is considered high quality, providing a detailed and comprehensive overview of the available evidence. According to the GRADE approach, the quality of the evidence and strength of the recommendations were rated as very low. Table S1 contains a summary of the findings and additional details.

## 4 Discussion

The key findings of this study are that an LFD is effective in reducing early satiety and bloating compared to controls but has minimal impact on nausea and may increase pain severity in some cases. In detail, early satiety decreased significantly and consistently across the studies, with no observed heterogeneity. Although challenge studies confirmed this result, they were less robust and exhibited high heterogeneity. Bloating decreased significantly in all RCTs but not in challenge studies. Both findings lacked robustness and exhibited high heterogeneity. Nausea did not change significantly in all RCTs and challenge studies, both sets of results were substantial and showed low heterogeneity. Pain increased significantly, though this result was not strong and showed moderate heterogeneity. Challenge studies confirmed this result, being robust but with high heterogeneity. However, the clinical relevance of these findings must be considered. Since symptom severity was generally assessed on a scale from 0 to 10, the WMD corresponds to relatively modest changes. Existing literature on dyspepsia suggests that the minimal clinically important difference (MCID) is around 1 point or 10%–15%. Consequently, the reduction in early satiety approaches clinical significance, while the reduction in bloating, despite being statistically significant, may have less clinical impact. Additionally, the small increase in abdominal pain is not likely to be of major clinical concern due to its minimal magnitude.

In NRSIs, bloating, nausea, and fullness significantly decreased following an LFD, but not pain. These findings were substantial but showed high heterogeneity. In this meta‐analysis, the certainty of all results was very low based on GRADE. Several findings, particularly for bloating and pain, were not robust, indicating a high sensitivity to the inclusion of specific studies. This suggests that the overall results may be driven by outliers or specific study conditions. These findings also exhibited high heterogeneity, with results varying significantly between different studies, underscoring the need for further research to accurately determine the impact of the LFD on dyspeptic symptoms.

As previously mentioned, an LFD has been proposed for managing GI symptoms. Numerous studies focusing on IBS patients have demonstrated the effectiveness of the initial phase of LFD in reducing symptoms such as abdominal pain, bloating, and flatulence [40, 42, 62, 63]. Since a considerable number of individuals suffer from both FD and IBS simultaneously, and these conditions share similar underlying pathogenic mechanisms [17, 64], the LFD holds potential as a treatment option for alleviating dyspeptic symptoms as well as its recognized benefits in IBS management [17].

Our meta‐analysis revealed that an LFD effectively alleviated bloating and early satiety, contrasting with some individual trials found in the systematic review [17, 18, 19]. For instance, in an RCT comparing wheat, rice, and mung bean, wheat ingestion for breakfast and lunch caused greater bloating and early satiety than rice and mung bean [18]. This difference may stem from the complete digestion and absorption of rice in the small intestine, whereas wheat is only partially digested and continues to the colon after lunch [65]. This process provides substrates for gut microbiota fermentation and gas production, contributing to greater bloating and early satiety [18]. Additionally, FODMAPs have a greater osmotic load, leading to increased water retention in the small or large intestine. This can result in abdominal distention and trigger bloating and early satiety [66], particularly in patients with visceral hypersensitivity [6]. However, the same study noted increased bloating with low‐FODMAP rice compared to high‐FODMAP mung beans [18]. This could be due to factors like timing of symptom assessment, intervention duration, individual differences (e.g., gut microbiota composition, specific sensitivities), and distinct impacts of various FODMAPs types (e.g., fructose, polyols). It also accounted for the quantity of FODMAPs consumed, non‐FODMAP elements (e.g., resistant starch in rice), and potential misclassification or contamination of studied foods [67]. Finally, the difference between the results of our meta‐analysis and some RCTs is likely due to the increased power of the meta‐analysis to identify smaller, but still potentially relevant, differences.

This systematic review identified that over 90% of RCTs reported that nausea had no significant change after LDF compared to control, as confirmed by the meta‐analysis. However, nausea significantly decreased when comparing pre‐ and post‐intervention scores, which results in a possible explanation: nausea is more likely caused by psychological rather than GI factors [68], suggesting that the placebo effect could influence symptom perception and that dietary interventions may not improve nausea.

In our systematic review, approximately 30% of studies reported significant pain reduction with an LFD compared to controls, likely due to reduced gas production and osmotic load, as previously discussed [66]. However, the majority of studies showed either no significant decrease or even a non‐significant increase in pain [15, 39]. Aggregating available data in a meta‐analysis initially showed a significant increase, which resolved upon excluding the study by Goyal et al. [15]. However, in challenge studies, the significant increase persisted regardless of study exclusion. Even in the meta‐analysis of NRSIs, LFD could not significantly reduce pain after intervention, which makes the most probable culprit the stress and anxiety associated with dietary restrictions, contributing to an increase in pain perception. Other explanations, such as differences in timing, duration, individual variations, FODMAPs types or quantities, and potential misclassification of foods, could also be contributing factors [67]. These findings, coupled with high heterogeneity, indicate that the studies differ in methods, populations, results, or other relevant factors, making it challenging to draw definitive conclusions.

NRSIs explore pre‐ and post‐intervention effects, assessing potential impacts, feasibility, and challenges, especially in early‐stage research. As FODMAPs investigation in dyspepsia is emerging, these studies guide future RCTs. We confirmed that NRSIs reveal challenging symptoms. NRSIs showed reduction in bloating, fullness, and nausea, but not pain. In the systematic review, the effect on pain varied by type, with no significant effect on idiopathic abdominal pain [47]. Less impact was found for early satiety [48] and persistent fullness [61].

Furthermore, NRSIs offer an overview of potential response rates, helping researchers set expectations for future participants during RCT recruitment. Our findings suggest that approximately three‐quarters of people are expected to respond to the intervention [56, 59]. This finding should be interpreted cautiously due to the observed high heterogeneity, and moderate to serious risks of bias.

In healthy subjects, variations in FODMAPs did not significantly impact dyspeptic symptoms, consistent with anticipated outcomes [36, 43, 52]. This suggests that the LFD may affect individuals differently based on their physiological susceptibility and does not inherently offer health benefits. Therefore, it should not be recommended for prevention in advance.

The LFD has several limitations, including inadequate nutrient intake, particularly calcium, which can negatively impact diet quality. It also alters the gut microbiota, potentially reducing beneficial bifidobacteria. Adherence to the LFD can be challenging and may not be achieved by everyone. Therefore, sustained adherence is generally not recommended [9]. However, the elimination phase of LFDs typically spans 6 to 8 weeks, followed by gradual reintroduction of specific FODMAPs while transitioning to a moderate FODMAP diet [69]. In this systematic review, only one study investigating the FODMAP reintroduction was identified [15]. The findings supported that once the three phases of the LFD are completed, symptom recurrence is less likely and quality of life is maintained, suggesting that long‐term adherence may be unnecessary [69].

Trials on FODMAP and GI symptoms should address additional confounding factors, including potential dietary triggers such as gluten, dairy products, and food chemicals, which require careful design considerations and measures to control for these variables [60]. Additionally, the restriction of gluten‐containing foods (such as wheat, barley, and rye) and dairy products in the LFD arm complicates identifying the specific component responsible for the observed effects [60]. This was not adequately addressed in the included studies in this review, necessitating caution in interpreting the results.

This study has limitations. First, variability among studies in populations, interventions, methodologies, and outcome measures introduced substantial heterogeneity, which prevented categorizing studies into specific groups. Second, due to the limited number of studies, subgroup analysis to explore the source of heterogeneity could not be performed. Third, diversity in baseline participant characteristics may have influenced results despite using a random‐effects model, potentially affecting the generalizability of findings. Fourth, most included studies had a moderate to high risk of bias, impacting the precision of findings. Fifth, estimates extracted from figures in some studies may lack precision. Sixths, some of the included studies had small sample sizes and short interventions, which affected the accuracy of our findings. Therefore, RCTs with larger sample sizes and longer duration are needed to determine the exact effect of FODMAPs on dyspeptic symptoms. Finally, the results were not sufficiently robust and were influenced by sensitivity analysis, necessitating a cautious interpretation of findings. Despite these limitations, the study possesses several strengths. A comprehensive literature search was conducted using diverse terms for dyspeptic symptoms and broad inclusion criteria to encompass all potentially relevant articles. Screening, data extraction, and quality assessment were independently conducted. Statistical analysis was performed on an adequate number of intervention arms, and subgroup analysis was conducted based on different designs, including LFD advice and FODMAP challenges. The study utilized the GRADE approach and was evaluated as a high‐quality review using the AMSTAR 2 tool. The majority of studies employed crossover designs, which reduce variability and increase statistical power.

In conclusion, we identified all clinical trials assessing the effect of FODMAPs on dyspeptic symptoms, irrespective of the participants' health conditions. Our findings suggest that an LFD can be an effective strategy for alleviating some FD symptoms, including early satiety and bloating, compared to controls. However, it has minimal impact on nausea and may increase pain severity. NRSIs also showed improvements in bloating, fullness, and nausea, but not pain. In healthy subjects, FODMAPs did not significantly impact dyspeptic symptoms and should not be recommended for prevention. However, these findings are considered to have very low certainty according to the GRADE assessment, which reduces confidence in the evidence. Given that current evidence is preliminary, and considering significant heterogeneity among studies, small sample sizes, and intervention variability, further well‐designed, large‐scale, long‐term RCTs are needed to clarify these results.

## Author Contributions

Peyman Adibi, Parisa Hajihashemi, and Fahimeh Haghighatdoost contributed to the design of the review protocol, defined the research theme, and screened the eligible studies. Seyedeh‐Zeynab Hosseinian, Parisa Hajihashemi, and Fahimeh Haghighatdoost contributed to screening the eligible studies, conducting the data extraction, and revising the article. Seyedeh‐Zeynab Hosseinian and Parisa Hajihashemi contributed to making the tables and writing the article. Peyman Adibi, Parisa Hajihashemi, and Fahimeh Haghighatdoost contributed to interpreting the results and providing expert advice on the article.

## Funding

The authors have nothing to report.

## Disclosure

All authors have read and approved the final version of the manuscript, Parisa Hajihashemi had full access to all of the data in this study and takes complete responsibility for the integrity of the data and the accuracy of the data analysis.

## Ethics Statement

The authors have nothing to report.

## Conflicts of Interest

The authors declare no conflicts of interest.

## Transparency Statement

Parisa Hajihashemi affirms that this manuscript is an honest, accurate, and transparent account of the study being reported; that no important aspects of the study have been omitted; and that any discrepancies from the study as planned have been explained.

## Supporting information

Supporting File.

## Data Availability

The data that support the findings of this study are available from the corresponding author upon reasonable request.
