# Supplementary material for: The Effects of Fermentable Oligo‐, Di‐, and Monosaccharides and Polyols on Dyspeptic Symptoms: A Systematic Review and Meta‐Analysis of Clinical Trials
Source: Health Sci Rep. 2026 Jun 18;9(6):e72662. doi: 10.1002/hsr2.72662 (PMC13277749; doi:10.1002/hsr2.72662)
Supplement: Supplementary file 1 — Supporting File. [file HSR2-9-e72662-s001.docx]

**Legend to figures:**

**Supplementary Figure 1.** Intervention arm of fermentable oligo-, di- and monosaccharides and polyols (FODMAP) on the severity of bloating in non- randomized studies of interventions (NRSI)

**Supplementary Figure 2.** Intervention arm of fermentable oligo-, di- and monosaccharides and polyols (FODMAP) on the severity of nausea in non- randomized studies of interventions (NRSI)

**Supplementary Figure 3.** Intervention arm of fermentable oligo-, di- and monosaccharides and polyols (FODMAP) on the severity of pain in non- randomized studies of interventions (NRSI)

**Supplementary Figure 4**. Intervention arm of fermentable oligo-, di- and monosaccharides and polyols (FODMAP) on the severity of fullness in non- randomized studies of interventions (NRSI)

**Overall (I-squared = 99.1%, p = 0.000)**

**Study (year)**

Valdez-Palomares (2021)

Chan (2020)

Peters (2016)

Yang (2021)

Peters (2016)

De Roest (2013)

-2.06 (-4.08, -0.05)

**ES (95% CI)**

5.81 (4.99, 6.63)

-1.91 (-2.15, -1.67)

-37.00 (-50.50, -23.50)

-4.00 (-4.29, -3.71)

-37.00 (-50.50, -23.50)

-2.00 (-2.22, -1.78)

100.00

**Weight (%)**

23.34

24.20

2.04

24.16

2.04

24.22

**-2.06 (-4.08, -0.05)**

5.81 (4.99, 6.63)

-1.91 (-2.15, -1.67)

-37.00 (-50.50, -23.50)

-4.00 (-4.29, -3.71)

-37.00 (-50.50, -23.50)

-2.00 (-2.22, -1.78)

0

-50.5

0

50.5

**Supplementary Figure 1-** Intervention arm of fermentable oligo-, di- and monosaccharides and polyols (FODMAP) on the severity of bloating in non- randomized studies of interventions (NRSI).

**Supplementary Figure 2-** **.** Intervention arm of fermentable oligo-, di- and monosaccharides and polyols (FODMAP) on the severity of nausea in non- randomized studies of interventions (NRSI).

**Overall (I-squared = 99.4%, p = 0.000)**

Frieling (2019)

Yang (2021)

Valdez-Palomares (2021)

Peters (2016)

Peters (2016)

**Study (year)**

De Roest (2013)

Chan (2020)

-1.15 (-2.13, -0.17)

-1.45 (-1.59, -1.30)

-2.50 (-2.71, -2.29)

0.04 (-0.00, 0.08)

-11.00 (-20.50, -1.50)

-11.00 (-20.50, -1.50)

**ES (95% CI)**

-1.00 (-1.18, -0.82)

0.17 (-0.21, 0.55)

100.00

19.71

19.61

19.79

1.01

1.01

**Weight (%)**

19.67

19.21

**-1.15 (-2.13, -0.17)**

-1.45 (-1.59, -1.30)

-2.50 (-2.71, -2.29)

0.04 (-0.00, 0.08)

-11.00 (-20.50, -1.50)

-11.00 (-20.50, -1.50)

-1.00 (-1.18, -0.82)

0.17 (-0.21, 0.55)

0

-20.5

0

20.5

**Supplementary Figure 3-** Intervention arm of fermentable oligo-, di- and monosaccharides and polyols (FODMAP) on the severity of pain in non- randomized studies of interventions (NRSI).

**Overall (I-squared = 98.8%, p = 0.000)**

Frieling (2019)

Yang (2021)

Valdez-Palomares (2021)

Peters (2016)

Peters (2016)

**Study (year)**

De Roest (2013)

Chan (2020)

**-1.04 (-2.26, 0.18)**

-1.07 (-1.20, -0.94)

-3.00 (-3.29, -2.71)

4.64 (3.95, 5.33)

-26.00 (-38.50, -13.50)

-30.00 (-42.00, -18.00)

**ES (95% CI)**

-2.00 (-2.23, -1.77)

-0.90 (-1.15, -0.65)

100.00

19.93

19.75

18.78

0.91

0.98

**Weight (%)**

19.84

19.81

0

-42

0

42

**Overall (I-squared = 98.4%, p = 0.000)**

Chan (2020)

Yang (2021)

De Roest (2013)

**Study (year)**

**-1.63 (-2.92, -0.35)**

-0.91 (-1.12, -0.70)

-3.00 (-3.32, -2.68)

-1.00 (-1.33, -0.67)

**ES (95% CI)**

100.00

33.60

33.23

33.17

**Weight (%)**

0

-3.32

0

3.32

**Supplementary Figure 4-** Intervention arm of fermentable oligo-, di- and monosaccharides and polyols (FODMAP) on the severity of fullness in non- randomized studies of interventions (NRSI).
